# Older Europeans’ experience of unmet health care during the COVID-19 pandemic (first wave)

**DOI:** 10.1186/s12913-022-07563-9

**Published:** 2022-02-12

**Authors:** Aida Isabel Tavares

**Affiliations:** 1 ISEG, Lisbon School of Economics and Management, Lisbon, Portugal; 2 CEISUC, Centre of Studies and Research in Health of the University of Coimbra, Coimbra, Portugal

**Keywords:** Unmet health care, COVID-19 pandemic, Europe, SHARE

## Abstract

**Background:**

During the COVID-19 pandemic the utilization of health services has changed. People were living in a very different social, economic and epidemiological context. Unmet health care is expected to happen. The purposes of this work are i) to compare the differences between unmet care across countries, ii) to find the main factors which are associated with unmet health care, which includes giving up and postponing medical care, as well as denial of medical care provision by the health services, and iii) to determine if health systems’ characteristics and government decisions on lockdown were related to unmet care.

**Methods:**

We have used the most recent dataset collected by the SHARE-COVID Survey during the summer of 2020. These data cover all EU countries and are applied to people over 50. We have estimated a set of logistic regressions to explain unmet health care.

**Results:**

The results indicate that women, people who are slightly younger, with higher education and income, who find it hard to make ends meet each month, and people with poorer health were more likely to experience unmet health care. We also found that in health systems with high out-of-pocket payments people are more likely to give up health care while in countries with previous high levels of unmet health needs this likelihood was the opposite; people in countries with a high number of beds per capita and with a Beveridge-type health system were reporting less postponement of health care.

**Conclusion:**

Some policy measures may be suggested such as social and economic measures to mitigate loss of income, expansion of the points and forms of access to health care to improve utilisation.

**Supplementary Information:**

The online version contains supplementary material available at 10.1186/s12913-022-07563-9.

## Background

Severe Acute Respiratory Syndrome Coronavirus 2 (SARS-CoV-2), generally known as COVID-19, erupted in China in December 2019, and soon it spread across the world. The first European case was reported in France on 24 January 2020 and four days later Germany was also reporting cases [1]. Quite soon, by 30 January, the World Health Organization (WHO) declared the outbreak of Coronavirus a public health emergency of international concern [2]. Since then, the spread of COVID-19 in Europe (and worldwide) has been exponential, as shown by Graph A 1[3], in the Additional file 1.

At the beginning of the outbreak not much was known about the virus, its effects and its transmission modes, but it became clear that it had a worse effect on older people. The risk of developing severe COVID-19 disease and serious complications was significantly higher for people older than 60 [4]. In fact, the COVID-19 pandemic’s impacts on older people were not restricted to the disease itself, a number of other impacts tended to hit as well. From family, social, and economic problems to health and psychological issues, everything seems to suffer [5, 6].

Europe has an ageing population [7] and governments had to respond to the fast and uncontrolled spread of COVID-19 which not only threatened a large percentage of the population, but pressured health systems, too, due to the sudden increase in demand for health care (mainly intensive care).

The response across countries resulted in many different measures taken by governments. These are clearly set out in the European Observatory COVID Response Monitor [8] and other publications [9]. These measures included lockdown and confinement, postponement of provision of healthcare services in order to accommodate COVID-19 patients, and a request for greater effort from the health sector workforce [10]. The impacts of the pandemic and the measures adopted to cope with it are severe and wide-reaching [11]. The reality in which people were living became very different from the one before the pandemic. People were facing social isolation, social distancing, and social disconnectedness; a controversy reality created by fake news [12]; and health systems highly focused on COVID cases.

One clear impact of the pandemic and the accompanying measures was the effect on access to healthcare services by older people [11]. Not only did they feel insecure and fear infection, but the services were unable to keep up with routines. So, there are several reasons why unmet health care demand and needs could be expected during the pandemic – because people renounced medical care, because such care was postponed, or because health services did not provide it. In other words, and from a wider perspective, there are motives both from the provision side of health care, such as providers lacking the conditions to look after non-covid patients, not enough health care professionals, and no physical space, and from the demand side like fear, no means of transport, compliance with lockdown measures, meagre household budget, self-assessment of the (non-)need for medical attention.

From a policy perspective, the pandemic situation forced some trade-off measures in the health system. The increased number of acute patients with COVID-19 who required increasing hospital resources. This redirection of resources meant that some non-COVID patients could not be looked after. So, some visits, diagnosis, treatment, and surgeries were cancelled and/or postponed. Moreover, the lockdown measures clearly conveyed the information that the situation was very serious. People felt these far-reaching measures, and some chose to relinquish medical attention, whether out of fear or to adjust to the situation, or for another personal reason.

This work sets out to compare differences in unmet health care in people aged over 50 across the EU during the first wave of COVID-19 pandemic, and to find the main factors associated with unmet health care. That is, what drives the unmet health care for older people, across European countries. It assesses not only the individual characteristics, but the macro-factors characterizing health systems, too, and the lockdown response to the pandemic decided by the governments. To achieve these aims we use data from SHARE COVID-19 Survey, which is the latest available data on this topic, covering all EU countries.

In this work, we focus on the demand for health care, which also includes the need for it, and we focus on the failure to satisfy that demand. We assume that demand is materialized in some action to look for health care services, either because there is need, or because there is another individual reason [13]. It is assumed that a need generates demand, but demand may not reflect a need. Lack of available data meant that we were not able to focus exclusively on unmet health needs since we have no data to isolate the health needs people may feel but that may not be fulfilled. So, we have taken a larger concept of demand for health care in the form of appointment or treatment. Unmet health care means that demand for health services was not satisfied, and this includes the situation where health needs were unmet. We assume that a large proportion of health care demand in fact corresponds to a need, and only a small proportion of this demand is the result of some desire that is not a need.

The literature on this topic during the pandemic has started to become available. Davillas and Jones [14] studied the UK case in 2020 using concentration indexes. They found that being female and suffering from chronic disease was associated with the cancellation of medical care; additionally, unmet needs were more pronounced in hospital care than in primary care, and they found a pro-rich inequality bias in the access to medical care. One study, conducted in Seoul, South Korea, during the first wave of the pandemic [15] found that women, young people, with lower levels of schooling, with white-collar jobs were more likely to experience unmet health care needs.

Two recent works based on SHARE COVID-19 data focused on the inequality of the effects of the pandemic on economically vulnerable people in Europe [16, 17]. The first study is based on the estimation of probits and concludes that the impact of economic vulnerability is notably stronger among those who were in poor health before the outbreak and the oldest. The authors of the second study, however, concluded that income-related horizontal inequity in unmet needs, pro-rich-type inequity, was not evident in most European countries according to the estimation of concentration indexes. Several authors [16–20] report that the economic downturn in the US, which resulted in a fall in income and higher unemployment, has made it more difficult to afford medical attention. In India, a study [21] based on a small-survey sample, concluded that about 23% of health needs there are unmet due to fear, lack of transport, or non-availability of healthcare services. Finally, a systematic review [22] of studies published up to 10 August 2020 concluded that health care utilisation decreased by about a third during the pandemic, with a great variation, and affecting more people with less severe illnesses.

Empirical literature about unmet health care (and needs) is usually based on micro-data so as to have information on individual characteristics. For Europe, and using European SILC-data and logistic regressions, a sample of people across Europe older over 16 years old, Chaupain-Guillot and Guillot [23] found that there was a positive relationship between the share of out-of-pocket payments in total health expenditure and the probability of finding unmet needs in a country. In an earlier work, using SHARE data for five European countries to estimate a set of multilevel logistic regressions, where the sample of respondents are older than 50, Mielck et al. [24] encountered the association between forgone care and income where the low-income groups are more likely to report forgone care than high income groups. A very recent work, and close to ours, also used data from SHARE COVID-19 to explain health care access and utilisation during the first wave of the pandemic [25]. They used both individual and institutional-country characteristics in a multilevel logistic regression with fixed slopes, despite the low level of intra-class correlations. Their findings point to higher unmet health care in (Western Europe) countries where the universal health coverage is wider and closure measures were stricter.

The concern with unmet health care, especially health needs, is not new and empirical studies have set out to find the main determinants (for instance, in 2006 a large report was presented for the European Union [26]). The basic conceptual modelling goes back to the late 1960s to Anderson [27, 28], who designed a framework explaining the demand for healthcare services [29]. Accordingly, the demand for health care is determined by the health system and the external environment, the population characteristics, including socioeconomic, community surroundings, and need. There is also an indirect influence coming from the health behaviour and health outcomes. The determinants of health, however, have been explained by [30] Whitehead and Dalhgreen (1991). These authors proposed a conceptual model which explains that individual health is determined partly by factors related to the socioeconomic and community environment, but also by other factors like the health system, and general socioeconomic, cultural and environmental conditions.

Our empirical analysis continues the line of previous work in this area of research. We use demographic, economic, health variables and macro-factors to explain the unmet health care, experienced by older people, in Europe, during the first wave of COVID-19. We base our modelling on the conceptual models by Dalhgreen and Whitehead and Anderson [27, 28, 30]. Thus, we consider individual characteristics which influence health outcomes while we also consider health system characteristics and the macro-context expressed by the lockdown measures imposed during the first wave of the pandemic.

First, we differentiate between the Beveridge and Bismarck type health systems. The Beveridge type tends to be a system financed by taxes and managed by the government whereas the Bismarck type is associated with social insurance and is managed autonomously by insurance organizations. Second, we distinguish health systems with high and low out-of-pocket payments, and we also distinguish those with high and low level of unmet health needs for people older than 65, in the year before the pandemic struck. We also account for the availability of health resources in the country, that is, doctors, nurses and beds per capita. Finally, we test the association of lockdown measures taken by several EU governments during the first wave of the pandemic on the different indicators of unmet health care. In this way, we are able to isolate the effects of this generally imposed measure and allow its association with reported unmet health care to be examined.

To sum up, unmet health care, both demanded and needed, can be largely explained by socio-economic and health status but also by contextual and macro-factors [27, 28]. Our work contributes to the discussion of this topic as it explores the factors that may explain the unmet health care in Europe during the first wave of COVID-19 pandemic. It imparts an analysis on common trends in the EU which could join in the currently evolving creation of the European Health Data Space and the debate about the European Health Union. Additionally, the results have been found to corroborate the increasing importance of e-health and telemedicine to overcoming unmet health care.

## Methods

### Population survey and sample

We used data collected by the Survey of Health, Ageing and Retirement in Europe (SHARE) [31, 32]. This is a multidisciplinary, cross-national panel database of microdata on health, socio-economic status and social and family networks of individuals aged 50 or older, not institutionalized. We have used the SHARE COVID-19 dataset released on 17 December 2020. Data was collected via Computer-Assisted Telephone Interviews (CATI) in the SHARE COVID-19 Survey between June and July 2020. Methodological issues for the data collection are available in Scherpenzeel et al. [33]. The full description, availability, and updates of SHARE are available on the project website (SHARE 2020) [34]. The SHARE dataset includes 25 EU countries as described in Table A1, in the Additional file 1. We have also used some data obtained previously in SHARE wave 7, as the number of chronic diseases. Our final sample accounts for 23,288 people.

### Empirical strategy

We begin by describing some statistics and then we estimate six logistic regressions. Three of these logistic regressions account for country controls and allow the analysis of individual factors; the other three logistic regression consider individual controls and allow the analysis of macro-controls.

The estimated model is given by the following equation:

$$Unmet\ {health\ care}_i={\beta}_0+{\beta}_1{Individual\ controls}_i+{\beta}_2 Macro-{controls}_i+{\varepsilon}_i$$

and

$$Unmet\ {health\ care}_i=\left\{\begin{array}{c}1\ if\ Unmet\ {health\ care}_i>0\\ {}0\ if\ Unmet\ {health\ care}_i\le 0\end{array}\right.$$

where *βs* are the parameters to be estimated and ε is the error term.

Estimated odds ratios (denoted by ‘or’) and average marginal effects (represented by dy/dx) are presented. The marginal effects are also referred to as difference in predictive margins and we use Stata command ‘margins, dydx(*)’, which gives the change in the probability of the dependent variable when there is a unitary change of the independent variable [35]. The *p-value* is presented for the odds-ratio; the *p-value* for the marginal effect is equal or differs by centesimal values from that one.

The estimations accommodate cluster countries heteroscedasticity and so variance-covariance estimators are clustered sandwich estimators. Additional testing is performed whenever possible. The VIF test is used to check multicollinearity across independent variables; the *linktest* is used for specification error; the pseudo-R^2^ and Wald test are used for testing the global significance of the model; Pearson and Hosmer-Lemwshow goodness of fit tests are also presented. All the empirical results were obtained using econometrics software STATA 15.

### Dependent variables for unmet health care

The dependent variables capture the unmet health care. There are three forms of these unmet needs and/or demand for health care services: giving up, postponing, or being denied access to medical care. These are obtained from three Yes-or-No questions as follows:

i) Since the outbreak of Corona, did you forgo medical treatment because you were afraid of becoming infected by the corona virus?
ii) Did you have a medical appointment scheduled which the doctor or medical facility decided to postpone due to Corona?
iii) Have you asked for an appointment for medical treatment since the outbreak of Corona and failed to get one?

These three questions reflect the demand for health care services, and they may include cases of unmet health care needs. However, they do not give sufficient information to disentangle demand from need.

Finally, we have considered an overall dependent variable which represents all forms of unmet health care.

### Independent variables for individual and macro-factors

We have described the independent variables in Table 1, which include individual and macro-factors or controls. We have grouped independent variables in demographic, economic, health, and finally, health system characteristics, and government response to the pandemic by means of lockdown measures.

i) Concerning the variable ‘dif_makends’, that is, difficulty to make ends meet. It is obtained from the survey question “Thinking of your household’s total monthly income since the outbreak of Corona virus, would you say that your household is able to make ends meet with great difficulty, with some difficulty, fairly easily, or easily?”. We have taken the answers ‘with great difficulty’ and ‘with some difficulty’ to mean that the household budget can barely cover expenses. This variable captures the difference between money revenue and household expenditures. So, people facing a short or negative difference will report finding ‘great’ or ‘some’ difficulty making ends meet. This is not a numerical variable but an ordered variable. One expects to find people with difficulties in making ends meet more likely to report unmet health care.

**Table 1:** Description of independent variables

Variables	Description
Demographic	
male	Dummy variable. Takes value 1 if male; 0 if female.
age	Number of years old in 2020.
education	Number of years of education.
Economic	
income	Natural logarithm of monthly household income per person before the pandemic. The survey question was “How much was the overall monthly income, after taxes and contributions, that your entire household had in a typical month before Corona broke out?”
dif_makends	Dummy variable. Takes value 1 if respondent says it is difficult to make ends meet with the household monthly income; 0 otherwise. The survey question was “Thinking of your household’s total monthly income since the outbreak of Corona, would you say that your household is able to make ends meet with great difficulty, with some difficulty, fairly easily, or easily.”
unemployment	Dummy variable. Takes value 1 if respondent is got unemployed during the pandemic; 0 otherwise. The survey question was “Due to the Corona crisis have you become unemployed, were laid off or had to close your business”.
Health	
SHA^−^	Self-assessed health before pandemic is a categorical variable. Ranges from 1 to 5, where 1 is excellent and 5 is poor. The survey question was “Before the outbreak of Corona, would you say your health was excellent, very good, good, fair, or poor?”
worse_health	Dummy variable. Takes value 1 if health got worse during the pandemic; 0 otherwise. The survey question was “If you compare your health with that before the outbreak of Corona, would you say your health has improved, worsened, or stayed about the same.”
chronic	Number of chronic diseases provided in Wave 7 of SHARE.
Health System	
beveridge	Dummy variable. Takes value 1 if health system is Beveridge type; 0 otherwise. Beveridge Health Systems: Sweden, Spain, Italy, Denmark, Portugal, Cyprus, Finland, Latvia, Malta.
high_OOP	Dummy variable. Takes value 1 if the level of Out-Of-Pocket payments are above the EU average in 2018; 0 otherwise. Countries with high OOP level are Bulgaria, Cyprus, Estonia, Greece, Hungary, Italy, Latvia, Lithuania, Malta, and Portugal. (Source Eurostat [36]).
high_unmetneeds	Dummy variable. Takes value 1 if the level of unmet health needs (no matter the reason) for people older than 65 is above the EU average in 2019; 0 otherwise. Countries with high level of unmet health needs are Estonia, Finland, Greece, Latvia, Poland, Romania, Slovakia and Slovenia. (Source Eurostat [36]).
high_doctors	Dummy variable. Takes value 1 if the number of doctors per 100,000 people is above the sample average in 2019 (or 2018 in case of missing value) equal to 233.43; 0 otherwise. Countries with high number of doctors: Bulgaria, Czechia, Denmark, France, Germany, Lithuania, Malta, Portugal, Spain. (Source Eurostat [36]).
high_nurses	Dummy variable. Takes value 1 if the number of nurses and midwives per 100,000 people is above the sample average in 2019 (or 2018 in case of missing value) equal to 463.12; 0 otherwise. Countries with high number of nurses and midwives: Belgium, Czechia, Cyprus, Denmark, Finland, France, Germany, Lithuania, Luxemburg, Malta, Slovakia, Switzerland (Source Eurostat [36]).
beds	Hospital beds per 100,000 people in 2019. Average number in the sample of countries is equal to 509.3. (Source Eurostat [36]).
Government Response	
no_lockdown	Dummy variable. Takes value 1 if government response to COVID-19 during the first wave of the pandemic in 2020 does not include a national lockdown; 0 otherwise. Countries with no lockdown: Malta, Latvia, Hungary and Sweden. This information is provided by Coronavirus Government Response Tracker [37].
Country controls	Set of dummy variables for each country.

On the other hand, the variable ‘income’ is the usual variable which is used to place a person in a socioeconomic status. People with high (low) income, have a high (low) socioeconomic status. This variable is not related to the previous one, because it only accounts for money revenue and it is a numerical variable. One expects that people with high (low) income will report unmet health care less (more) often. This variable was adjusted to purchasing power parities using the conversion rates provided by the SHARE survey. We have used the current rates for 2017 and Germany takes value 1 as the reference country.

ii) The variables concerning health, that is, ‘Self-assessed health (SAH)’, ‘Worse health’ and ‘Chronic diseases’ are controls we have available for potential need. All these three variables are interpreted as their value increases, health gets worse. It is expected that people with lower self-assessed health status, with a deteriorating health status or suffering from chronic disease are more likely to be in need of care or to demand more health care. We are not able to predict the sign for these independent variables, it could be positive or negative, depending on the outcome experienced by the individual.
iii) When the variable “high_OPP” takes value 1, it represents countries with a level of out-of-pocket payments above the EU average. This variable captures the level of universality and the access to health care in the country. Countries with high levels of OPP are expected to register higher levels of unmet health needs.
iv) The variable “high_unmet” represents countries with a level of unmet health needs by people older than 65 in 2019 in Europe, that is, before the pandemic. In the Additional file 1, Graph A2 shows the percentage of self-reported unmet needs for medical examination for people aged 65 years or over. In this way, it becomes possible to identify the relationship between the previous normal scenario and the one during the pandemic first wave.
v) The variables “high_doctors”, “high_nurses” and “beds” capture the health resources available in the country. Both “high_doctors”and “high_nurses” represent countries with a number of professionals per 100,000 people above the average of the countries sample. The variable “beds” measures the number of hospital beds per capita in each country.
vi) We have considered the dummy variable ‘beveridge’ so that health systems are differentiated according to their main source of financing. This is relevant because the reforms introduced in Beveridge type health systems aimed reducing waiting times and increasing patient choice, while Bismarck type health system reforms aimed controlling the costs supported by insurance companies paid to providers [38]. Additionally, some research indicates differences in patient satisfaction between Beveridge and Bismarck health systems [39]. For these two reasons, we found it relevant to include this independent variable.
vii) Lastly, some comments concerning the variable “no_lockdown”. The concept of lockdown is not defined by WHO. So, we take it as a general measure used by governments during the pandemic. A general definition was provided by Mboera et al. [40] and it includes: (i) geographical containment; (ii) home confinement; and (iii) the closure of social, educational and economic activities, and prohibition of mass gatherings. For the purpose of our analysis, we have considered the information provided by the Corona virus Government Response Tracker [37] to identify the countries that did not impose such restrictive measures during the first wave at a national level.

## Results

### Descriptive statistics

**Fig. 1:**
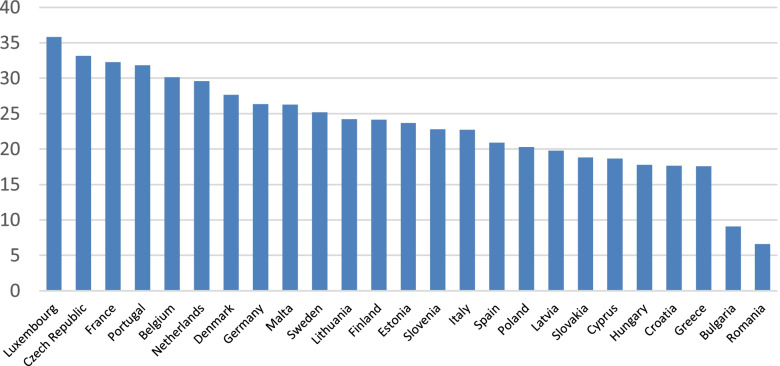
Percentage of people, aged 50+, reporting unmet health care during first wave of the pandemic in EU (SHARE)

The percentage of people in the EU over 50 years old reporting unmet health care during the pandemic varies widely from country to country, as displayed in Fig. 1. For instance, in Luxembourg, we find just over 35% of people reporting unmet health care, while in Romania the figure is five times lower, at about 7%. The distribution of the percentages of people reporting some sort of unmet care during the first wave of the pandemic (Fig. 1) does not follow the same pattern as that found in the distribution of the percentage of people over 65 reporting unmet health needs across Europe in 2019 (Graph A2, in Additional file 1).

**Fig. 2:**
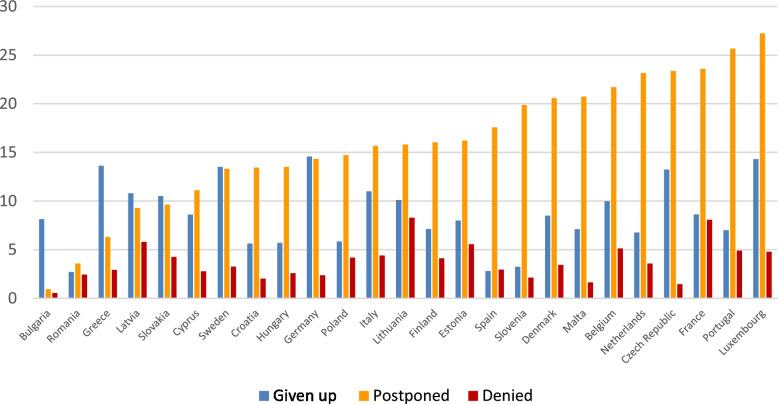
Percentage of people, aged 50+, reporting different forms of unmet health care during first wave of the pandemics in EU

The reasons for unmet health needs across the EU countries are also different and there is no single pattern, as we can see in Fig. 2. While Bulgaria has the lowest percentages of people reporting unmet health care explained by postponing or denial, the highest figure for postponing is found in Luxembourg and the highest percentage of people being denied access to health care is found in Lithuania. As for giving up medical care, the lowest percentage giving this answer is found in Spain and highest is in Germany.

The remaining descriptive statistics are presented in Table 2, where both the percentage of respondents and the mean value for independent variables are displayed. The majority of the respondents are women, the average age is 71 years, and the average number of years of schooling is about 11. Around 3% of respondents (that is, 800 people) became unemployed after the beginning of the pandemic and more than 37% report difficulties making ends meet. The mean monthly value of the per capita household income, adjusted by purchasing power parity, is almost €1000. More than a third of the respondents consider that their health is fair or poor, slightly more than 9% felt that their health has got worse during the pandemic; the average number of chronic diseases reported is two and about 19% of respondents had no chronic illness.

**Table 2:** Brief descriptive statistics

	%respondents		mean
male	35.95	age (years old)	71.00
unemployment	3.44	education (years)	11.29
difficulty to make ends meet (dif_meetends)	37.77	chronic diseases (number)	1.95
worse health	9.23		
fair or poor health (SHA)	34.58		
*N* = 23,288			

### Logistic regression results

We estimated six logistic regressions to find the main associated factors for unmet health care in EU countries for people aged 50+. The odds ratios and marginal effects corresponding to these estimates are presented in Tables 3 and 4.

**Table 3:** Estimated results for unmet health care – individual factors

	Given up			Postponed			Denied		
	or	dy/dx		or	dy/dx		or	dy/dx	
male	0.651	−0.044	***	0.892	−0.019	***	0.964	−0.002	
age	0.993	−0.001	**	0.989	−0.002	***	0.978	−0.001	***
education	1.028	0.003	***	1.021	0.004	***	1.016	0.001	
income	1.174	0.017	***	1.163	0.026	***	1.109	0.005	
dif_makends	1.334	0.032	***	1.020	0.003		1.100	0.005	
unemployment	1.147	0.015		1.112	0.018		1.069	0.004	
SHA	1.243	0.023	***	1.154	0.024	***	1.310	0.014	***
worse_ health	1.554	0.053	***	1.185	0.030	***	2.100	0.048	***
chronic	1.096	0.010	***	1.115	0.018	***	1.124	0.006	***
_cons	0.031		***	0.058		***	0.017		***
country controls	yes			yes			yes		
Number of obs	23,281			23,281			23,281		
Pseudo R2	0.048			0.053			0.059		
linktest	significant			significant			significant		
Pearson chi2	23,169.20 (0.541)			23,119.89 (0.630)			22,968.07 (0.851)		
Hosmer-Lemeshow chi2	5.11 (0.746)			12.43 (0.133)			11.66 (0.167)		

Note: _cons (constant) estimates baseline odds; ***p-value < 0.01, ** *p*-value < 0.05; dy/dx for factor levels is the discrete change from the base level

**Table 4:** Estimated results for unmet health care – macro-factors

	Given up			Postponed			Denied		
	or	dy/dx		or	dy/dx		or	dy/dx	
beveridge	0.750	−0.030		0.600	− 0.083	*	0.659	−0.020	
high_OOP	1.432	0.041	*	0.812	−0.035		1.220	0.010	
high_unmetneeds	0.691	−0.039	***	0.652	−0.072	***	0.894	−0.006	
high_doctors	0.969	−0.003		0.965	−0.006		0.953	−0.002	
high_nurses	1.499	0.044	**	1.186	0.030		1.506	0.021	**
beds	0.999	0.000		0.998	0.000	**	0.998	0.000	
no_lockdown	1.100	0.010		0.821	−0.033		1.016	0.001	
_cons	0.69		***	1.046			0.049		***
individual controls	yes			yes			yes		
Number of obs	23,281			23,281			23,281		
Wald chi2	2590.61(0.000)			784.10(0.000)			1086.90(0.000)		
Pseudo R2	0.0318			0.0284			0.0424		
linktest	not significant			not significant			significant		
Pearson chi2	22,952.09 (0.883)			23,059.03 (0.755)			23,174.43 (0.561)		
Hosmer-Lemeshow chi2	26.69 (0.000)			44.31 (0.000)			5.02 (0.756)		

Note: _cons (constant) estimates baseline odds; ***p-value < 0.01, ** *p*-value < 0.05, * *p*-value < 0.10; dy/dx for factor levels is the discrete change from the base level

The results obtained for VIF test are shown in Table A3 in the Additional file 1. Since all the VIF values are under 10, there is no potential multicollinearity across independent variables.

We begin by describing the results shown in Table 3 and focus our attention on the individual factors.

First, demographic factors show that being male and being older decreases the likelihood of reporting an unmet health care while education increases it. The exception goes for the logistic regression explaining denied health care, for which only age is statistically significant.

Second, results relating to economic factors are diverse. We found that people with higher incomes are more likely to report given up or postponed health care. However, people with difficulty in making ends meet also tend to report more often giving up health care. For instance, someone reporting difficulty in making ends meet is about 33% more likely to report this type of unmet health care. We did not find statistical significance for this variable in the other two logistic regressions. Additionally, we did not find either statistical significance for becoming unemployed during the pandemic.

Third, all three health indicators show that worsening health means that people are more likely to report unmet health care. People who found their health was getting worse during the pandemic are about 55% more likely to have given up unmet care, 18.5% more likely to report postponement, and twice the people were more likely to report denied health care, comparing with those people who did not report a worse health. People with worse health status and with chronic disease are also reporting unmet health care more often than those in better health.

We next turn our attention to the results presented in Table 4 concerning the macro-factors associated with the unmet health care.

Our findings show that Beveridge health systems only matter favourably in postponed health care. People in these countries were reporting postponed care less often. People in countries featured by high OOP report having given up health care about 43% more often, while people in countries characterized by high levels of unmet health needs were less likely to reply having either given up or postponed health care. The high number of nurses contributes to finding people who have given up or postponed health care. Finally, the number of beds is not significant in two of the regressions; it is statistically significant in the regression explaining postponed health care, but the magnitude of the effect is about null. Nevertheless, as an example, focusing on this regression and plotting the statistically significant marginal effects we obtain Graph A4 (in the Additional file 1). It is shown that as the number of beds increases, the predicted decrease in postponed health care in Beveridge health systems and countries with high levels of unmet health care needs becomes smaller.

## Discussion

Life changed during the COVID-19 pandemic. Governments have imposed several measures to mitigate the effects on the health system of the sudden enormous increase in the demand and need for healthcare. These measures had a big impact on every single part of society. The impacts on older people were significant. Not only were they more prone to suffer from serious COVID-19 illness, but also they faced social isolation, where fear was a common feeling. In this context, unmet health care, demand and needs, are expected to be prevalent among older people because they have given up or postponed accessing medical care, or because of the unavailability of medical care in the health system.

Our work aimed to compare the differences in unmet health care across EU countries and to find its main determinants or associated factors. We have analysed the influence of individual factors and of macro-factors related to the health system and with the lockdown response. We used the most recent statistical information collected by SHARE-COVID for people older than 50 in the EU during the summer of 2020. This survey clearly frames the questions under the Corona-virus pandemic to mitigate against any framing bias among respondents.

The first group of relevant results shows that being female, younger, with higher levels of education, higher income, and difficulty in making ends meet each month can increase the likelihood of reporting unmet health care; additionally, as health gets worse or if it is already in a poorer state, the more likely it is to experience unmet health care.

The second group of relevant results shows that in Beveridge health systems the odds of reporting health care postponement are lower; in countries where people face high levels of OPP, it is more likely to find people giving up health care; in countries which have been characterized by high level of unmet health needs of older people, we find lower probabilities of unmet health care; and finally, higher number of nurses per capita, increased the likelihood of giving up healthcare and having it denied.

Let us first compare our results with those found previously in a pre-pandemic context and focused on unmet health needs, because this was the beginning of the *status quo* (and the information available). For this comparison, we will be focusing mainly in the individual factors. The main drivers of unmet health needs in the EU based on data from SILC and SHARE are similar to some extent to those we have found now. We should mention that the SILC survey includes Europeans older than 16, in a non-pandemic context, while the SHARE survey includes people older than 50 (before and during the pandemic). This is an overall comparison because the frameworks of the surveys are very different, nevertheless we think the information provided by SILC is valid and it could work as reference.

Concerning demographic characteristics, results based on SHARE survey [24], before the pandemic, found that women were more likely to report unmet health needs, as we did found; the findings obtained from SILC survey [23] found no gender effect. But women tend to seek medical care more often than men [41, 42] so it is more likely to find women reporting unmet healthcare needs. Age was found to be negatively related to unmet health needs in Chaupain-Guillot and Guillot [23], as we did, but Mielck et al. [24] did not find such relation.

Higher education is usually related to higher levels of unmet health needs, as found before [23] in a non-pandemic context and as we found for the giving up and health care postponement. People with higher levels of education, tend to give up medical attention more often. In a pandemic context, it could be that people with more education tend to look for information in other sources, like the Internet, scientific articles, social networks and media, or from friends. May be high educated people made a pro-social decision by considering that releasing health care resources, it would benefit the well-functioning of health care systems. Or it could also be that they have a higher opportunity cost for availing themselves of medical care and consequently they tend to give up medical care more often because the risk of getting COVID would be too high compared to the risk of not having health care. It is interesting to note that we did not find a significant correlation between education and denial of medical care. This may also show that people with higher levels of education, who have more resources (cognitive, communicative, relational) would carry out an information collection and only look for medical help in serious and acute situations.

When it comes to people’s economic characteristics the results are diverse. As expected from both SILC and SHARE surveys [22, 23], higher incomes are associated with lower unmet health needs, despite this may not be so linear and clear in some countries [23]. However, we found a different relationship where higher incomes are associated with higher probability of giving up and postponing health care. Perhaps the opportunity cost of getting health care during the pandemic is higher for people with higher incomes; may be these people can afford to look for alternatives to health care soon, or may be people with higher incomes, have better health status so they do not need so much health care [43]. This result seems to go against the existing trend of economic inequalities for health care access and utilisation. However, we also found partial evidence of these inequalities [44–47]. People who have difficulties in making ends meet each month are more likely to report giving up medical care. This might be expected to happen during first wave of the pandemic at the beginning of the social and economic crisis, when people would give up medical attention because the household budget had to be directed to other expenditure.

Concerning health status characteristics, our results are identical to what has been found previously in a pre-pandemic context [22, 23]. People with worse health are those more likely to report unmet needs, because of their health status and their higher level of need. The pandemic has very likely contributed to aggravating this situation because people fear infection and would rather stay at home than go to a healthcare unit. On the other hand, healthcare services are more likely to deny care to people with not-so-good health status (lower health status, worsening health and suffering from chronic diseases), because of the pressure on providers to attend to COVID patients, and also to prevent potential contagion with a serious disease.

Now, to compare our results with other (as yet scarce) evidence collected during the pandemic. First, concerning individual older people drivers in Europe for unmet health care, studies show that there is a general trend where women, people with difficulties making ends meet, and people whose health is poor, tend to experience this more often [15, 17, 18, 22], which coincides with our findings. Older people are also very likely to report unmet health care [25] but this finding is less clear in other studies [16, 17] and we found a negative but not expressive relationship between age and unmet health care.

Income and education are also far from being a consensual result. While we found that higher incomes were associated with higher levels of unmet health care, this was not found in all European countries [17] or it did not present a significant result on some occasions [16]. Before the pandemic, higher incomes are associated with lower unmet health care because people use their resources to access health care. During a pandemic, economic resources can be used differently, in particular, they might be saved for using in the near future, as the pandemic gets controlled. On the other hand, education before the pandemic was associated with more unmet health care, as it is during the pandemic in some countries [17]. Or it may not even be significant, or even be negative [15]. Education gives people instruments to assess and judge the situation and make decisions. But what this pandemic has shown is that too much information, whether contradictory, fake, or well-supported, can impact people differently even for the same level of education. So a high level of education could in fact be associated with lower use of health care.

It is found as a general trend where there is a reinforcement or persistence of health inequalities [14–22, 41, 42, 44–47]. Not only people with lower economic resources but also those with weaker health are more likely to report unmet health care [16, 22, 23, 44]. This general trend depreciates social capital but also erodes social cohesion. Moreover, in the future, the degree of severity of illnesses will be higher, more treatments will be demanded, and this will imply higher health expenditure or maybe more premature deaths.

Secondly, concerning macro-factors related to the health system and to the lockdown environment people were living in, scarce evidence exists in the literature. A recent study [24] found no effect of the lockdown measures decided by the government on health care being given up by older people, as we found, but a significant effect was found for postponing, which we did not confirm. Giving up or postponing health care may not be related to lockdown measures. This could happen because medical care or treatment was accounted for as a special situation under which lockdown measures would not apply. This means that people would not feel the institutional pressure to avoid medical care.

Unlike that previous study, which found no evidence concerning the type of health system, we did find that in countries with a Beveridge-type of health system, health care was less often postponed. One possible reason for the difference in these results is the controls considered in the model. Once other characteristics of the health system have been considered, such as the importance of OOP, unmet health needs and health resources, different results may appear.

We have also found that people in countries characterised by high OOP were giving up health care more often, most likely as a saving mechanism for the uncertain future created by the pandemic. On the other hand, in countries with high levels of unmet health care needs before the pandemic, lower levels of unmet health care were reported. This could be explained as a securing behaviour from people to guarantee access which otherwise could not be used.

An overview of the influence of the macro-factors related to the health system characteristics might ultimately conclude that on the whole they did not significantly impact unmet health care during the first wave of the pandemic for people older than 50. There are some instances of concern which may deserve future research. For instance, Bismark-type health systems seemed to be more prone to postpone health care and countries with a high number of nurses registered higher odds of given up and denied health care. Despite these instances and the structural differences across European health systems, it may be worth examining the explanations of unmet health care and other health system outcomes based on micro-factors related to organizations, such as care and teams’ organization, leadership, how health professionals are paid and the strategies they adopted to cope with difficult work conditions.

One important limitation on data is the absence of information concerning the type and intensity of unmet health care (especially the needs), that is, we have no information about the number of times the situation occurred, and we have no information about the type of health care that was relinquished, nor the need felt by the person, nor if the health care was under private insurance. Future research could well focus on finding possible associations between the unmet care and the diseases reported by people.

Another limitation is related to the survey. On the one hand, the questions on unmet health care have not been tested before. On the other hand, there could be some bias, whether selection, attrition, or reporting bias. However, this bias can operate in either direction by underestimation or overestimation of the true effect, and at the end, accounting for all the potential bias, there is no bias in the results. Future meta-analysis will be able to measure this potential bias.

The final limitation is the impossibility of considering all potential factors that are associated with unmet health care in Europe. For instance, health equipment, information systems, and other technology are certainly factors that mattered during the pandemic and we have not included them in our controls because the information is either not enough or not reliable. Or because it would go beyond the purpose of this analysis and potentiate future research.

It is important to note that SHARE data was collected between June and July 2020, that is, in the summer when the first COVID-19 wave was fading (as observable in Additional file 1 Graph A1). The second wave started in the autumn, with numbers escalating and economic difficulties growing significantly across all European countries. We think that our results will be more pronounced as family budgets decline, fear persists, and medical care continues to be cancelled. Additional studies will follow as more statistical data is released, as it will for SHARE COVID new wave.

## Conclusions

Several policy recommendations can be derived from our work. First, social and economic measures to mitigate loss of income are needed, but this is a widely recognised purpose of the current policy. Then, the relationship between poorer health and higher unmet health care could be attenuated by policies aimed at expanding the points of access to healthcare, in particular to primary healthcare to ensure that attention is given to those suffering from chronic diseases. These include the provision of e-health and teleconsultations [48] at home or in community points such as parish council offices and community centres; integrated community pharmacies with primary healthcare units that could facilitate prescriptions and home deliveries of drugs for people suffering from chronic diseases; social home visits by community and/or police force teams to older people to relieve social isolation and increase the likelihood of health care. Measures strongly aimed at the socioeconomic factors and to the individual needs are those with a strongest health impact [49].

From the point of view of the health system, we found that there is no single characteristic that properly explains all the unmet health care experienced during the first wave of the pandemic. In fact, health systems are very diverse in their structure and general characteristics and there are other very relevant features at micro-level that contribute to the performance of the health system and the expectations of people. Issues like how care and teams are organized, how leadership is activated, the way health professionals cope with difficulties, and the way health care units support the mental health of professionals, do influence and make a difference in the observed health outcomes. Unfortunately, international comparisons are difficult to perform at this level, but a European Horizon Research Project could embrace this sort of purpose. Health systems are going through several reforms [50] and more are expected after the experience brought by the pandemic. They will eventually be assessed.

Finally, this work should add to the current discussion on two European topics: the European Health Data Space and the European Health Union [51]. The first one will enable country comparisons and the identification of cases of better practices, and the second one points to the need to strive for the health and wellbeing of all Europeans [52].

## Supplementary Information

**Additional file 1.**

## Data Availability

Data are publicly available in SHARE project at http://www.share-project.org/. Data availability is public and free upon registration.
