# Supplementary material for: Older Europeans’ experience of unmet health care during the COVID-19 pandemic (first wave)
Source: BMC Health Serv Res. 2022 Feb 12;22:182. doi: 10.1186/s12913-022-07563-9 (PMC8840072; doi:10.1186/s12913-022-07563-9)
Supplement: Supplementary file 1 — Additional file 1. [file 12913_2022_7563_MOESM1_ESM.docx]

**Older Europeans’ experience of unmet health care during the COVID-19 pandemic (first wave)**

APPENDIX

Graph A1: Daily new confirmed COVID-19 cases per million people (7-day rolling average)


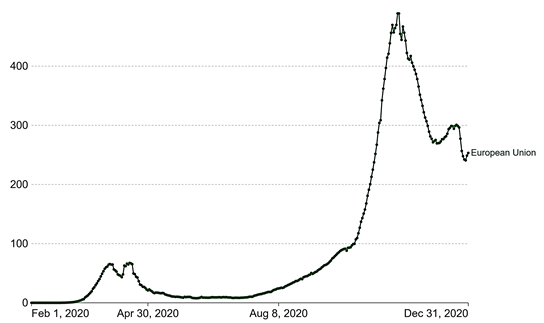


Source: Our World in Data (2021); Raw data from COVID-19 Data Repository by the Center for Systems Science and Engineering (CSSE) at Johns Hopkins University.

Graph A2: Percentage of self-reported unmet needs for medical examination for 65 years or over, 2019 (Eurostat)

Graph A3: Percentage of people, aged 50+, reporting unmet health needs during first wave of the pandemics in EU (SHARE)

Table A1: Countries and respondents

| 1 | Belgium | 5148 | 9 | France | 2552 | 17 | Malta | 1211 |
| --- | --- | --- | --- | --- | --- | --- | --- | --- |
| 2 | Bulgaria | 1068 | 10 | Germany | 3113 | 18 | Netherlands | 946 |
| 3 | Croatia | 2997 | 11 | Greece | 4651 | 19 | Poland | 477 |
| 4 | Cyprus | 1115 | 12 | Hungary | 1385 | 20 | Portugal | 1652 |
| 5 | Czechia | 3353 | 13 | Italy | 5031 | 21 | Romania | 2325 |
| 6 | Denmark | 2593 | 14 | Latvia | 1259 | 22 | Slovakia | 1245 |
| 7 | Estonia | 5726 | 15 | Lithuania | 1701 | 23 | Slovenia | 4436 |
| 8 | Finland | 1726 | 16 | Luxembourg | 1377 | 24 | Spain | 2851 |
|  |  |  |  |  |  | 25 | Sweden | 1652 |

Table A2: Reported unmet health care across countries

|  | Given Up | Postponed | Denied |
| --- | --- | --- | --- |
| Germany | 451 | 443 | 74 |
| Sweden | 223 | 220 | 54 |
| Netherlands | 64 | 219 | 34 |
| Spain | 80 | 501 | 84 |
| Italy | 552 | 785 | 222 |
| France | 218 | 602 | 204 |
| Denmark | 219 | 532 | 86 |
| Greece | 632 | 292 | 135 |
| Belgium | 513 | 1108 | 262 |
| Czechia | 443 | 782 | 49 |
| Poland | 278 | 700 | 197 |
| Luxembourg | 197 | 375 | 66 |
| Hungary | 79 | 187 | 36 |
| Portugal | 115 | 421 | 80 |
| Slovenia | 144 | 881 | 94 |
| Estonia | 457 | 925 | 316 |
| Croatia | 169 | 400 | 61 |
| Lithuania | 171 | 269 | 141 |
| Bulgaria | 84 | 9 | 6 |
| Cyprus | 96 | 123 | 31 |
| Finland | 122 | 275 | 71 |
| Latvia | 133 | 116 | 73 |
| Malta | 85 | 250 | 20 |
| Romania | 63 | 83 | 57 |
| Slovakia | 131 | 120 | 53 |
| Total unmet care | 5,719 | 10,618 | 2,506 |

Table A3: VIF test

| Variable | VIF | 1/VIF |
| --- | --- | --- |
| beveridge | 3.64 | 0.274619 |
| beds | 2.88 | 0.347277 |
| high_nurses | 2.76 | 0.362079 |
| high_ doctors | 2.19 | 0.457569 |
| high_OOP | 1.65 | 0.607015 |
| no_ lockdown | 1.50 | 0.667601 |
| high_unmetneeds | 1.44 | 0.692135 |
| SHA | 1.39 | 0.720347 |
| income | 1.38 | 0.723613 |
| dif_makends | 1.28 | 0.779516 |
| education | 1.26 | 0.793809 |
| age | 1.24 | 0.805153 |
| chronic | 1.24 | 0.806002 |
| worse_health | 1.05 | 0.952321 |
| uemployment | 1.05 | 0.956133 |
| gender | 1.03 | 0.971955 |
| Mean VIF | 1.69 |  |

Graph A4: Marginal effects for postponed care
